# Is Seed and Soil Theory Suitable for Metastatic Spread of Pituitary Carcinomas?

**DOI:** 10.3389/fendo.2020.607405

**Published:** 2021-01-05

**Authors:** Congxin Dai, Bowen Sun, Jun Kang

**Affiliations:** Department of Neurosurgery, Beijing Tongren Hospital, Capital Medical University, Beijing, China

**Keywords:** pituitary carcinoma, metastatic spread, seed and soil theory, systemical spread, craniospinal spread

Pituitary carcinomas (PCs) are defined as tumors of adenohypophyseal origin that show craniospinal and/or systemic metastases (1). PCs are extremely rare, making up only 0.1% to 0.2% of all pituitary tumors, but represent a challenge to clinical practice (2). Although research on PCs has progressed rapidly, the pathogenesis and biology of pituitary carcinomas have not yet been well understood due to their rarity. Although some hypotheses have been postulated, they remain controversial. To date, the patterns of metastatic spread in PCs are complex and not yet fully elucidated, needing further research.

The “seed and soil” theory of metastasis was proposed by Paget in 1889. Based on analysis of autopsies in 735 fatal cases of breast cancer, Paget suggested that the spread of metastatic cells was organ-specific and not merely anatomical and involved interaction between the cancer cells and the host organ and that metastases developed only when the seed and soil were compatible (3). After proposal of the “seed and soil” theory, it was validated and supported by abundant animal studies (4–6). In the past 130 years, it has been proven that organ-specific patterns of metastases exist in many cancers (7). However, the concept of “seed and soil” has not yet been discussed in the metastasis of PCs thus far. The purpose of this commentary is to provide a brief perspective on our understanding of the mechanisms of metastasis of PCs and discuss whether the “seed and soil” theory is suitable for PCs.

Currently, there are three possible mechanisms of metastatic spread in PCs, including drop metastasis (occurrence in surgical tract), blood-borne dissemination, and central venous dissemination (8). Although tumor cells dropping from the surgical site and drifting *via* the cerebrospinal fluid pathway occurs mostly during surgery, overall PCs tend toward systemic metastasis rather than craniospinal metastasis. The frequency of systemic metastasis is approximately 47% in overall PCs, the frequency of craniospinal metastases in PCs is approximately 40%, and the other 13% of PCs exhibit both (9). Most PCs (88%) are functional and commonly include adrenocorticotropic hormone (ACTH)- (42%) and prolactin (PRL)-secreting tumors (33%), whereas different types of PCs prefer different patterns of metastatic spread. In one series, PRL-secreting PCs spread systemically more often (71%) than ACTH-secreting PCs (57%); in contrast, growth hormone (GH)-secreting carcinomas present more commonly with cerebrospinal metastasis (10). The sites of PC metastasis include the cerebral cortex, cerebellum, spinal cord, leptomeninges, eyes, heart, lung, cervical lymph nodes, pancreas, liver, kidney, pelvic lymph nodes, ovary, myometrium, and bone. The most common locations of metastasis are extracranial or intracranial and spinal spread, but the preferential specific site for metastasis remains uncertain due to the limited number of reported cases (11).

The “seed and soil” theory was proposed based on large-scale autopsy of patients with fatal breast cancer, whereas PCs are very rare tumors, with only 170 cases reported in the literature (9). Thus, it is difficult to verify whether this theory is suitable for metastatic spread of PCs *via* large-scale clinical case analysis. However, we can obtain some useful information from limited cases and surmise some possible conclusions.

First, the vast majority of reported PCs are endocrinologically active, including ACTH-, and PRL-secreting carcinomas, which are the most common. Although dural invasion is most frequently found in nonfunctioning PAs at 54.2%, the incidence of nonfunctioning PCs is very low (12). A literature review of PCs reported that the most common tumors were ACTH-secreting (34.7%), PRL-secreting (23.6%), and Null Cell (15.3%) respectively (13). In this review, the most common site of metastasis in total 25 cases of ACTH-secreting PCs were intra-cranial, liver, and spinal respectively, whereas the most common site of metastasis in 17 cases of PRL-secreting PCs was intra-cranial, lymph nodes, and bones respectively. This review indicated more CNS metastases noted in the cases reviewed than systemic metastases. However, the other previous reviews reported that systemic metastases appear more common than CNS metastases in PCs (9, 14). Another large retrospective series also reported that ACTH-secreting and PRL-secreting PCs showed a greater tendency toward systemic metastasis than central nervous system metastasis (10). In addition to intra-cranial, the metastasis of ACTH-secreting carcinomas has a predilection for the liver and spinal (10, 15, 16), and the metastasis of PRL-secreting carcinomas exhibits preferences for lymph nodes and bones (9–11, 16, 17). A possible explanation is that the different hormone-secreting tumor cells have their own suitable microenvironment and favorable host organ, thus selectively invading compatible organs and growing in a proper environment. Therefore, these results indicate that the spread of metastatic PC cells is organ-specific and involves interactions between cancer cells and the host organ, not merely through the anatomic structure. However, this conclusion needs to be further validated by more cases of PCs and animal studies.

Second, almost all PCs present initially with an invasive pituitary macroadenoma, and metastatic PC cells have also been isolated from both cerebrospinal and pleural effusion fluid (9). Interestingly, most PCs tend to disseminate systemically *via* hematogenous and lymphatic spread rather than *via* craniospinal spread. In addition, there is no genetic, molecular, or histologic distinction between PCs and invasive or aggressive PAs. Moreover, the PRL-secreting tumors transformed to PCs twice as fast as the ACTH-secreting tumors (4.7 vs 9.5 years, respectively) (10).

Therefore, these data demonstrate that the process of metastasis starts with the release of malignant cells (seed) with metastatic potential from primary tumors, and different types of metastatic PC cells need different durations of metastatic latency to invade favorable host tissues (soil).

Together, these data support that different types of PCs prefer different patterns of metastatic spread and have their own favorable host organs. Therefore, as a viable mechanism, the seed and soil theory may be suitable for the metastatic spread of PCs. However, this conclusion was obtained based on limited cases of PCs reported in the literature and needs to be validated and supported by more cases and animal studies.

## Author Contributions

CD and BS drafted the manuscript and carried out the literature search. JK reviewed the manuscript. All authors contributed to the article and approved the submitted version.

## Conflict of Interest

The authors declare that the research was conducted in the absence of any commercial or financial relationships that could be construed as a potential conflict of interest.

The reviewer CY declared a shared affiliation with the authors to the handling editor at time of review.

## Abbreviations

PCs, pituitary carcinomas; PAs, pituitary adenomas; ACTH, adrenocorticotropic hormone; PRL, prolactin; GH, growth hormone.
